# Relationship between Changes of Nasal Length and Upper Lip Height during Smile

**Published:** 2016-09

**Authors:** Arash Beiraghi-Toosi, Seyed Mohammad Motamedalshariati, Samira Ghanei, Rowshanak Afshar

**Affiliations:** 1 Surgical Oncology Research Center, Mashhad University of Medical Sciences, Mashhad, Iran;; 2 Vascular and Endovascular Surgery Research Center, Mashhad University of Medical Sciences, Iran;; 3 Student Research Committee, Faculty of Medicine, Mashhad University of Medical Sciences, Mashhad, Iran

**Keywords:** Depressor septi nasi, Nasal length, Upper lip height, Dynamic rhinoplasty, Smile

## Introduction

Depressor septi nasi is an important muscle in the nose and upper lip dynamics.^1^^-^^4^ Hypertrophy of this muscle leads to a deformity during smiling presented by depression of nasal tip, shortening of upper lip, and a horizontal crease in upper lip.^4^ Rhino-gingivolabial syndrome was defined as depression of nasal tip, shortening of upper lip, and gingival show. This syndrome was attributed to the hypertrophy of the depressor septi nasi muscle.^5^ In cases with depressor septi nasi hypertrophy, varied combinations of smiling deformity components are encountered. This raises the question about coincidence of hyperactivity of different parts of this muscle.^5^

Some cases have isolated nasal lengthening, some have isolated upper labial shortening, and some cases presented the combination. If there is a relationship between nasal lengthening and upper lip shortening, resection of depressor septi nasi (DSN) in patients with DSN hypertrophy during rhinoplasty may affect upper lip changes during smile. In this study, nasal length and upper lip changes during smile are studied.^5^ The results of this study can help predict changes of the upper lip during smile following manipulation of DSN.

**Fig. 1:**
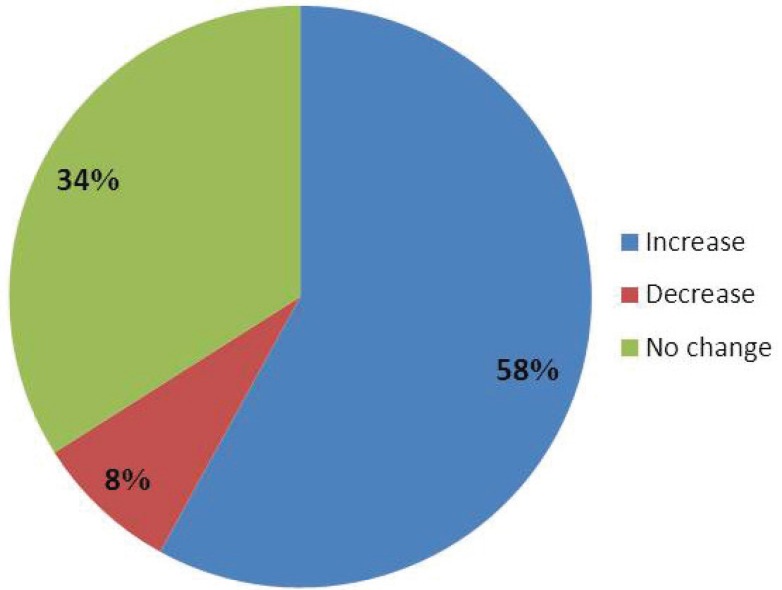
Measurements on lateral photographs in the repose and maximum posed smile

Fifty cases were enrolled. Exclusion criteria were age less than 18 years, history of operation in the nasal or lip area, and cleft lip. Photographies were done with digital camera with maximum zoom from a distance that the forehead hairline to the chin was visible. Standard photography was done with forward gaze and neutral neck position in standing, in lateral position, in the repose and maximum posed smile. Nasal length was measured from nasion to tip of the nose. Upper lip height was determined from subnasale (the point of the columella to lip attachment) to labrale superior (the midline point of the lip skin attachment to the projecting point of the nasal tip). To obviate possible errors due to changes of photograph size, the height of the middle third of the face from glabella to subnasale was measured for standardization of measurements (Figure 1). Statistical Analysis was performed by the SPSS software 11.5.

**Fig. 2:**
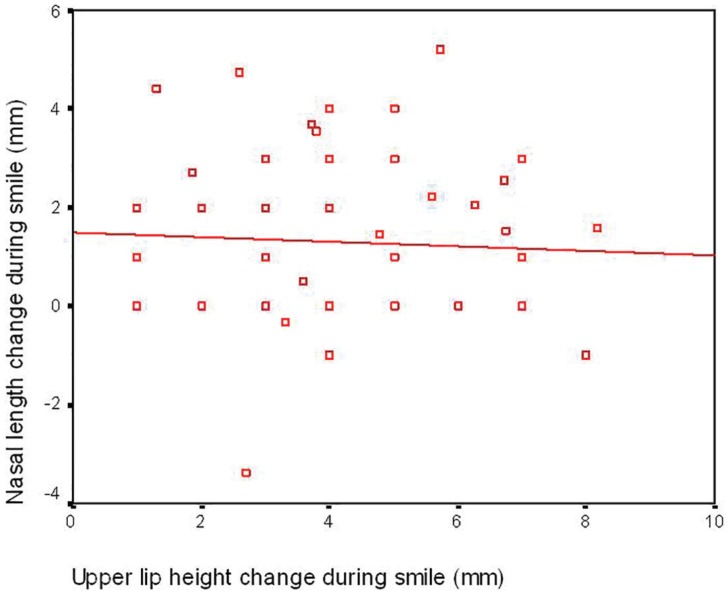
Nasal length change during smile

In this study, 46 cases (92%) were female and 4 cases (8%) were male. the mean age was 26±5.45 (min=18, max=37 years). The mean change in nasal length during smile was 1.31±727 mm increase of length. In 29 cases (58%), nasal length was increased during smile; 4 cases (8%) demonstrated decrease of nasal length during smile and 17 cases (34%) had no change in nasal length with smile (Figure 2). The maximum increase of nasal length during smile was 5.19 mm and the maximum decrease was 3.39 mm. Considering the percentage of the change, the mean increase of nasal length during smile was 2.53±3.35 percent. The maximum increase was 12.07% and the maximum decrease of nasal length was 6.16%. 

**Fig. 3:**
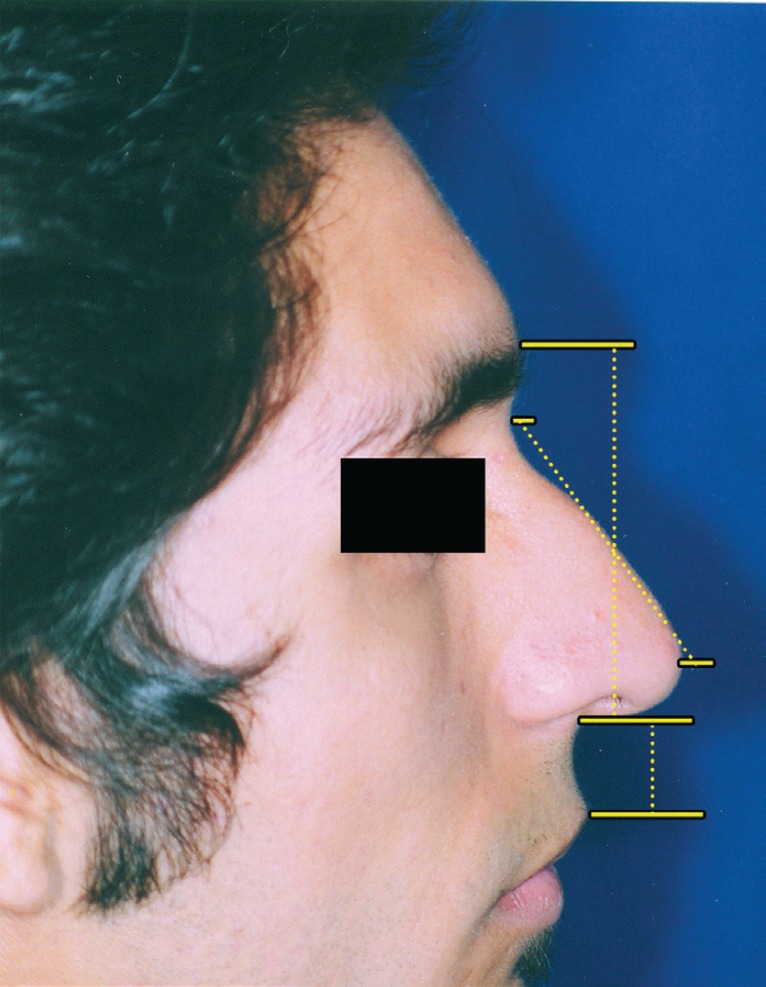
Correlation between nasal length and upper lip height changes during smile

The mean decrease of upper lip height during smile was 4.2±1.921 mm. The maximum decrease in height was 8.17 mm and the least decrease was 1 mm. Upper lip height decreased in all patients. The mean percentage of decrease of upper lip height was 30.37±12.610 percent (max=52.11%, min=7.14%). There was no statistically significant correlation between nasal length and upper lip height changes during smile (Pearson=0.054, df=48, *p*=0.712) (Figure 3). There was no relationship between the percentages of the measurements. Upper lip change during smile was not significantly different between those with increased nasal length (hyperactive DSN group) and those without increased nasal length during smile (t=0.575, df=48, *p*=0.568). 

Depressor septinasi is active in some people and rudimentary in others. Hypertrophy of this muscle leads to a deformity during smile that is consisted of three parts including (i) elevation of upper lip, (ii) a horizontal groove in upper lip and (iii) descending nasal tip. In the Rohrichstudy on the anatomy of DSN muscle, 16% of cases had rudimentary or undetectable muscle.^4^ In one study, 17% of cases had rudimentary or float muscle or fibrous tissue replacement.^6^ In another study, 36% of rhinoplasty candidates had hyperdynamic nasal tip related to DSN muscle. Their clinical criteria for hyperdynamic tip were decreasing nasal tip projection, elevation and shortening of upper lip, and increasing gingival show.^2^

In our study, upper lip height and nasal length were studied as components of smiling deformity. In all cases, upper lip was elevated during smile but it does not mean that all cases had DSN hyperactivity because in only 58% of cases, descending of nasal tip was present. It seems that descending of nasal tip during smile is a more reliable criterion for DSN hyperactivity. Several muscles act on the upper lip dynamics. In our study, no significant relationship was observed between nasal length and upper lip changes during smile. If we consider DSN hyperactivity as increasing nasal length during smile; upper lip height changes during smile would not be related to DSN hyperactivity. Or, different actions of DSN and its effects on nasal tip and upper lip may be independent.

DSN was described as a digastrics muscle consisting of columellar and labial parts and an intermediate tendon that attaches to the anterior nasal spine. These two parts may have dependent or independent functions.^7^ This finding is consistent with our results. Various components of smiling deformity may be independent and this must be considered in the studies evaluating DSN hypertrophy. A surprising finding in our study was that cases with nasal lengthening during smile had less shortening of upper lip. Although this was not statistically significant, this supports the independent action of columellar and labial parts of DSN.

This study can mean that manipulation of DSN during rhinoplasty will not affect upper lip height change during smile following operation. Confirmation of this assumption needs more clinical studies. Effects of DSN manipulation during rhinoplasty and analysis of different criteria of the smiling deformity due to DSN hyperactivity are among the subjects for future studies.^7^ It was shown that cutting the superior part of the orbicularis oris muscle following DSN repositioning to treat smiling deformity, orbicularis oris would affect smiling deformity due to relationships with DSN.^8^ It seems that DSN manipulation is effective on nasal tip changes during smile not the whole components of smiling deformity. Upper lip component requires other manipulations such as cutting the upper part of the orbicularis muscle. Besides, we should consider that in some anatomic variants, DSN muscle may attach to the periosteum instead of orbicularis oris muscle.^4^^,^^6^

DSN hypertrophy is considered as a reason for shortness of the columella in Negroid population.^9^ Our study implies that further studies for discrimination of various components of DSN hypertrophy in Negroid ethnicity are required to support or reject this theory. The relationship of nasal tip and upper lip was studied and various types of people were described,^1^ while these findings are in consistent with the results of our study. It seems that we should reconsider the DSN hypertrophy definition. In future studies, we should discriminate between nasal tip and upper lip changes during smile for determination of DSN hypertrophy. The smiling deformity should be divided to the nasal tip and upper lip components and its description should be revised.
